# Editorial: Current advances in genomics and gene editing tools for crop improvement in a changing climate scenario

**DOI:** 10.3389/fgene.2023.1214679

**Published:** 2023-06-12

**Authors:** Vijay Rani Rajpal, Deepmala Sehgal, Ravi Valluru, Sukhwinder Singh

**Affiliations:** ^1^Depatment of Botany, Hansraj College, University of Delhi, Delhi, India; ^2^ Syngenta, Jeolett’s Hill International Research Center, Bracknell, United Kingdom; ^3^ University of Lincoln, Lincoln, United Kingdom; ^4^ USDA-ARS, SHRS, Coral Gables, FL, United States

**Keywords:** genomics, gene editing, crop improvement, abiotic stress, disease resistance, climate change, crop productivity

## Introduction

The unprecedented global climate change has severely impacted our environment and engendered severe threats to agricultural productivity (Shahzad et al., 2021; Cinner et al., 2022; Ozdemir, 2022). This has led to the emergence of new races of plant pathogens and insect pests, accentuated abiotic stresses, depleted water resources and shrunken arable land, posing grave challenges to the food security of an ever-increasing global population (IPCC Sixth Assessment Report, 2022). The advantages offered by the Green Revolution of the mid-1960s are also fading away, resulting in a fragile food system (Davis et al., 2019; John and Babu 2021). Agriculture today faces newer challenges exacerbated by genetic erosion, the narrow genetic base of commercial crops and environmental degradation. There is an urgent need to make agriculture more resilient and sustainable while still continuing to develop high-yielding, stress-resistant and climate-smart crop varieties.

The advancements in genomics and gene editing technologies have offered immense opportunities and potential solutions for the genetic improvement of crops (Gao 2021). A plethora of novel avenues opened by genomics and genome editing approaches are attributed to the evolution of valuable tools like next-generation sequencing (NGS) methods, state-of-the-art genotyping arrays, genome mapping and genomic selection technologies that have helped to expedite the crop breeding processes. Likewise, new gene editing platforms have allowed precise editing of agronomically important genes to generate new varieties with desired characteristics (Zhang 2020; Ahmad 2023).

The deployment of these technologies has laid down the foundation of modern breeding for effectively channelizing the underutilized diversity hidden in the crop wild relatives into elite gene pools (Sehgal et al., 2015; 2017; 2020; Singh et al., 2018; Singh et al., 2021). The advanced breeding programs are assisted by cutting-edge genomics, sequencing and genome editing technologies and have integrated artificial intelligence, machine learning bioinformatics and other related disciplines to meet the global food demands (Huang et al., 2022; Robert et al., 2022; Ahmad 2023).

In this Research Topic, we have collated a total of 19 articles, including original research and review articles, to get an essence of the spectrum of current efforts undertaken by applying modern tools for crop trait improvement. To develop a background on the theme, we have included three review articles entitled “Comprehending the evolution of gene-editing platforms for crop trait improvement” by Dhakate et al., “Advances in crop breeding through precision genome editing” by Nerkar et al. and “Integrating CRISPR-Cas and next-generation sequencing in plant virology” by Mushtaq et al. that have very well built up the narrative of the Research Topic. The other crop-specific comprehensive reviews entitled “CRISPR for accelerating genetic gains in under-utilized crops of the drylands: progress and prospects” by Sharma et al., “Recent advances in date palm genomics-a comprehensive review” by Rahman et al., “Unclasping potentials of genomics and gene editing in chickpea to fight climate change and global hunger threat” by Singh et al., “A CRISPR way for accelerated improvement of cereal crops” by Basu et al., “CRISPR/Cas genome editing system and its application in potato” by Zhang et al., “Physiological and molecular basis of drought and heat stress tolerance to enhance productivity and nutritional quality of peanuts in harsh environments” by Puppala et al., “A perspective on selectable marker-free genome engineered rice: past, present and future scientific realm” by Singh et al. and “CRISPR/Cas genome editing in potato: current status and future” by Tiwari et al. provide an up-to-date detailed compilation of the published research in date palm, dry-land crops, peanuts, chickpea, potato, and rice.

There has been continuous evolution in the development of gene editing-based technologies involving CRISPR/Cas platforms. Traditionally used CRISPR/Cas nucleases followed Sequence-Specific Nucleases (SSNs) such as Zinc-Finger Nucleases (ZFNs) and Transcription Activator-Like Effector Nucleases (TALENs), and led to domains such as epigenome editing, base editing, and prime editing. Dhakate et al., Tiwari et al. and Basu et al. have comprehensively reviewed the evolution of CRISPR/Cas systems into new-age methods of genome engineering across various plant species and the impact that they have had on tweaking plant genomes and associated outcomes on crop improvement initiatives. Hou et al. reviewed the four types of CRISPR/Cas structures and mechanisms available today and the application of CRISPR/Cas9 systems in overcoming the challenges of self-incompatibility and improving the quality and resistance of potato. The review describes how precise knocking or targeted mutagenesis of *S-RNase* and *Sli* genes using CRISPR/Cas9 has helped to create self-compatible and self-incompatible potatoes, respectively. Additionally, the suitability of CRISPR/Cas9 and CRISPR/Cas13a systems in knocking out more than 22 genes has been detailed. Kor et al. have described the role of RNA Pol III promoters in precisely targeting genetic variants in genome editing. Nerkar et al. has focused on the advancements in crop breeding through precision genome editing. This review has included an overview of different breeding approaches for agronomic traits such as disease resistance, abiotic stress tolerance, herbicide tolerance, yield, and quality improvement, reduction of anti-nutrients, improved shelf life; genome editing tools and approaches used for crop improvement and an update on the regulatory approval of the genome-edited crops. Sharma et al. has described the opportunities of implementing genome editing technologies in under-utilized crops to increase genetic gains.

Zang et al. generated *Nud* mutants in wheat (*TaNud*) and barley (*HvNud*) using CRISPR/Cas9-based gene editing and investigated the heritability of the mutations in wheat. *Nud* gene is a transcription factor controlling the formation of the caryopsis, and loss-of-function mutation in the gene leads to the naked hull phenotype in barley. With the latest CRISPR/Cas9-based gene editing, combined with PCR-RE (polymerase chain reaction-restriction enzyme) approach, Zang et al. achieved a high editing efficiency (51.7%) of the three *Nud* alleles/homologs in wheat. This study has proven that with the improved vector system and CRISPR/Cas technology, it is not difficult to achieve precise genetic modification even in complex polyploid crops such as wheat which remained calcitrant to genetic modification technologies for a long time.

Heat and drought stresses cause substantial yield losses to crop production. According to the latest estimates, the heatwave episodes will particularly intensify in the Indo-Gangetic plains of India, which supports rice–wheat cropping system (Krishnan et al., 2020). Simulation models have predicted a reduction in rice yield by 10% for every 1°C rise in ambient temperature (Peng et al., 2004; Mendez et al., 2021). Breeding heat tolerant rice varieties is therefore a major objective of Indian breeders. Vast genetic variation has been reported to be present for reproductive stage heat stress (RSHT) tolerance in rice, however, modern genetics and genomics approaches have not been utilized fully to explore this variation. Ravikiran et al. comprehensively investigated genetic variation in RSHT tolerance with the GWAS approach using the cutting-edge genotyping arrays available in rice. They utilized three GWAS models to identify significant marker-trait associations (MTAs) for spikelet fertility and grain yield. Most significantly, the authors reported a set of stable MTAs for both traits, showing an advantage of 6–10 g for yield and up to 28% for spikelet fertility. Additionally, they identified promising tolerant genotypes that carried favorable alleles of 29, 28, 25, or 24 putative MTAs, which could serve as new donors in nurseries.

Peanuts exposed to drought stress at the reproductive stage are prone to aflatoxin contamination, which imposes a restriction on the use of peanuts as health food and also adversely impacts the peanut trade (Masaka et al., 2022). The review by Puppala et al. focuses on the significant progress that has been made towards the characterization of germplasm for drought and heat stresses tolerance and identifying MTAs and QTLs associated with drought tolerance. Advances in phenomics and artificial intelligence to accelerate the timely and cost-effective collection of phenotyping data in large germplasm/breeding populations are also reviewed and discussed. A holistic breeding approach that considers drought and heat-tolerant traits to simultaneously address both stresses is introduced as a successful strategy to produce climate-resilient peanut genotypes with improved nutritional quality.

Kumar et al. reported development of bread wheat variety HD3411 following marker-assisted backcross breeding for drought tolerance. They reportedly transferred four drought tolerance quantitative trait loci (QTLs) controlling traits, *viz.* canopy temperature, normalized difference vegetative index, chlorophyll content, and grain yield from the drought-tolerant donor line, C306 into a popular high-yielding, drought-sensitive variety HD2733. Marker-assisted selection coupled with stringent phenotypic screening was used to develop a promising genotype, HD3411. The line HD3411 has shown higher yield over selected cultivars and has been identified for varietal release and testing in the northeastern plain zone of the wheat-growing region in India.

Besides abiotic stresses, plant disease outbreaks threaten global food security significantly. The crop pathogens are responsible for a substantial reduction in global crop yield and productivity. The global burden of viral, bacterial and fungal pathogens in farmers’ fields ranges between 20% and 30% (Savary et al., 2019). It poses grave challenges, such as the emergence, spread and evolution of novel and more virulent races. Further, to address the challenges of crop loss due to various pathogens, improved disease surveillance and detection methods along with developing disease-resistant varieties are extremely important. Mushtaq et al. have shown that the NGS coupled with CRISPR-based genome editing have enabled rapid engineering of resistance by directly targeting specific genomic sites of plant viruses and viroids. They also discussed the emerging developments in NGS technologies and CRISPR/Cas-based DNA or RNA tests for the characterization of plant viruses along with their potential advantages and limitations. Kaur et al. employed BSA-seq approach in a wild species of rice *Oryza glaberrima* and identified a novel locus on chromosome 6 for resistance to root-knot nematode (*Meloidogyne graminicola*). They reported 3 potential candidate genes within QTLs qNR6.1, qNR6.2 and qNR2.1. This research has expanded the breadth of genes available for resistance to root-knot nematode and the possibilities of deploying new genes in rice breeding.

Machine learning (ML) and artificial intelligence (AI) algorithms have been increasingly used nowadays to improvise genomic prediction models to predict the phenotypes of newly developed breeding lines. Montesinos-López et al. in the article “A general-purpose machine learning R library for sparse kernels methods (SKM) with an application for genome-based prediction” demonstrated the usefulness of six machine learning models based on sparse kernel algorithm in two major crops, wheat and maize. Most importantly, this new package with six models allows user to use different formats of data, i.e., binary, categorical, count and continuous response variables. Recently, ML models are also being explored to resolve the off-target problems associated with CRISPR-Cas9. An article on ‘Machine learning in the estimation of CRISPR-Cas9 cleavage sites for plant system’ by Das et al. developed models on three ML-based techniques to estimate the cleavage sites of a given sgRNA. Out of the 11 trained models, the models based on the random forest technique, Artificial Neural Networks (ANN1-ReLU) and Support Vector Machine (SVM-Linear), performed better in the estimation of CRISPR-Cas9 cleavage sites showing an accuracy of 96.27%**.**

Breeding for soft-seeded varieties is an important objective in pomegranate (*Punica granatum* L.) breeding programs. The genetic architecture of seed-type trait has not been investigated much using modern approaches, and this has hampered the development of farmer-preferred and commercially viable pomegranate varieties. The recent advances in whole genome sequencing and transcriptome profiling in pomegranate have opened vistas for large-scale discovery of markers for trait discovery and improvement. A previous study in pomegranate had identified novel pre-miRNAs for seed-type trait in pomegranate (Luo et al., 2018). However, PCR-based markers were not generated in the study. The comprehensive study by Patil et al. utilized 761 potential novel pre-miRNAs to identify SSRs and generate breeder-friendly markers for use in breeding. They identified 227 and 79 SSR motifs specific to 60 pri-miRNA and 65 pre-miRNA sequences, respectively, and mapped them on the Tunisia genome. Most importantly, using target prediction and network analysis they reported the association of five miRNA-SSRs i.e., miRNA_SH_SSR35, miRNA_SH_SSR36, miRNA_SH_SSR53, miRNA_SH_SSR69 and miRNA_SH_SSR103 with seed type trait, which could be deployed by pomegranate breeders.

To summarize, a diverse collection of research and review articles included in this Research Topic has generated valuable information on the development of genetic and genomic resources in a variety of cereals (wheat, barley and rice), legumes (chickpea and peanut), fruit (pomegranate and date palm) and underutilized dryland crops. While, the review articles presented information on the evolution and refinement of new-age genomics, genome editing, and genome prediction models based on ML and AI algorithms for crop improvement, the research articles involved their application culminating into disease resistant, drought and heat resistant, high yielding crop varieties for instance line HD3411 in wheat (Kumar et al.). We believe that the articles compiled in this Research Topic will expand the existing knowledge on the strategies of crop improvement to mitigate climate change and ensure future food security.
